# Sulfur Dioxide: An Emerging Signaling Molecule in Plants

**DOI:** 10.3389/fpls.2022.891626

**Published:** 2022-05-09

**Authors:** Zhong-Guang Li, Xiao-Er Li, Hong-Yan Chen

**Affiliations:** ^1^School of Life Sciences, Yunnan Normal University, Kunming, China; ^2^Engineering Research Center of Sustainable Development and Utilization of Biomass Energy, Ministry of Education, Kunming, China; ^3^Key Laboratory of Biomass Energy and Environmental Biotechnology, Yunnan Normal University, Kunming, China

**Keywords:** sulfur dioxide, signaling molecule, seed germination, fruit fresh-keeping, stomatal movement, stress response

## Abstract

Sulfur dioxide (SO_2_) has long been viewed as toxic gas and air pollutant, but now is being verified as a signaling molecule in mammalian cells. SO_2_ can be endogenously produced and rapidly transformed into sulfur-containing compounds (e.g., hydrogen sulfide, cysteine, methionine, glutathione, glucosinolate, and phytochelatin) to maintain its homeostasis in plant cells. Exogenous application of SO_2_ in the form of gas or solution can trigger the expression of thousands of genes. The physiological functions of these genes are involved in the antioxidant defense, osmotic adjustment, and synthesis of stress proteins, secondary metabolites, and plant hormones, thus modulating numerous plant physiological processes. The modulated physiological processes by SO_2_ are implicated in seed germination, stomatal action, postharvest physiology, and plant response to environmental stresses. However, the review on the signaling role of SO_2_ in plants is little. In this review, the anabolism and catabolism of SO_2_ in plants were summarized. In addition, the signaling role of SO_2_ in seed germination, stomatal movement, fruit fresh-keeping, and plant response to environmental stresses (including drought, cold, heavy metal, and pathogen stresses) was discussed. Finally, the research direction of SO_2_ in plants is also proposed.

## Introduction

For so long, sulfur dioxide (SO_2_) has been viewed as a harmful gas and air pollutant. SO_2_ can dissolve in water and form sulfurous acid (H_2_SO_3_), which in turn dissociates into sulfite ( ${\mathrm{SO}}_{3}^{2-}$ ), bisulfite ( ${\mathrm{HSO}}_{3^{-}}$ ), and hydrogen ion (H^+^). In neutral solution, the ratio of ${\mathrm{SO}}_{3}^{2-}$ / ${\mathrm{HSO}}_{3^{-}}$ is 3/1 (M/M; Singh et al., 2012). Therefore, the toxicity of SO_2_ mainly roots in three derivants, that is, ${\mathrm{SO}}_{3}^{2-}$ , ${\mathrm{HSO}}_{3^{-}}$ , and H^+^. ${\mathrm{SO}}_{3}^{2-}$ and ${\mathrm{HSO}}_{3^{-}}$ are strong nucleophiles, which can deleteriously react with the sulfhydryl groups (–SH)-containing proteins (including enzymes) and then alter their functions and activities, thus disturbing physiological and biochemical metabolism (e.g., photosynthesis, respiration, and ion balance), and even leading to cell death. H^+^ can lower the pH value of the cells and their compartmentations, followed by affecting the activities of the enzymes and interfering with the cellular metabolism (Huang et al., 2021). In addition, SO_2_ can induce the accumulation of reactive oxygen species (ROS) by oxidizing sulfite into sulfate and/or activating NADPH oxidase (NOX), which in turn cause biomembrane damage, protein disintegration, DNA damage, chromosome aberration, gene mutation, Golgi body destruction, and programmed cell death (PCD) (Okpodu et al., 1996). Therefore, high concentration of SO_2_ can damage plant at morphological, physiological, biochemical, and molecular levels. For example, SO_2_ can reduce photosynthesis by disrupting thylakoid function, interfering with electron transport chain and membrane permeability, destroying pigments, and affecting carbon allocation, leads to leaf damage (e.g., yellow spots, discoloration, and necrosis), growth inhibition, and even plant death (Lee et al., 2017; Huang et al., 2021; Li and Yi, 2022).

SO_2_, as a signaling molecule, can endogenously produce by the catalysis of aspartate aminotransferase (AAT) and NOX using hydrogen sulfide (H_2_S) as substrate in animal system (Huang et al., 2021). Endogenous SO_2_ executes a positive role in reducing brain injury, lung injury, myocardial injury, and hypertension; regulating vascular remodeling, myocardial remodeling, collagen remodeling, ion channels, cell proliferation, endoplasmic reticulum stress, and protein posttranslational modification (mainly sulfenylation by H_2_O_2_). Therefore, the abnormal production of endogenous SO_2_ commonly leads to colitis, hypertension, atherosclerosis, neuronal damage, vascular calcification, myocardial hypertrophy, myocardial injury, pulmonary hypertension, and acute lung injury (Huang et al., 2021). These studies indicate that SO_2_, as signaling molecule, plays an essential role in many physiological and pathological processes.

In general, signaling molecules, such as calcium ion (Ca^2+^), ROS, nitric oxide (NO), and H_2_S, exhibit the common characteristics: small molecule, fast dispersal, dual effects, controllability of generation and elimination, biological activity, reprogramming gene expression, and so forth (Chen et al., 2011; Khan et al., 2019). Small molecules are easy to be quickly biosynthesized/released to initiate signaling, and then immediately eliminated to terminate signaling. In addition, signaling molecules can be rapidly spread from production sites to effect sites to exert their biological effects by their receptors/sensors. Dual effects of signaling molecules refer to their toxicity at high concentration and signaling role at low concentration. Therefore, they must be maintained homeostasis in cells (Khan et al., 2019). Finally, signaling molecules can specially bind to their receptors/sensors and then reprogram gene expression, further regulating cellular metabolism (Chen et al., 2011). It is quite clear that small molecule SO_2_ meets these criterions, indicating its signaling role in organisms.

In plants, SO_2_ can be generated in the form of ${\mathrm{SO}}_{3}^{2-}$ during sulfate $\left({\mathrm{SO}}_{4}^{2-}\right)$ reduction (Li et al., 2020). Recently, exogenous application of SO_2_ in the form of gas (mg m^−3^) or solution (Na_2_SO_3_/NaHSO_3_, 3/1, M/M), at low concentrations, has been found to exert a positive role in seed germination (Wang et al., 2017; Sun et al., 2018; Guo et al., 2021), stomatal movement (Taylor et al., 1981; Wei et al., 2013; Hu et al., 2014; Yi et al., 2017), fruit fresh-keeping (Joradol et al., 2019; Zhang et al., 2022), and plant response to adverse environments (Hu et al., 2015; Zhu et al., 2015; Han et al., 2018, 2019; Xue and Yi, 2018; Li et al., 2021; Ma, 2021). These studies indicate that SO_2_ is emerging as a signaling molecule in plants. However, the review on SO_2_ signaling in plants is little summarized. In this review, SO_2_ homeostasis in plants and its signaling role in seed germination, stomatal action, fruit fresh-keeping, and plant response to environmental stress (including drought, cold, heavy metal, and pathogen infection) were concluded. The aim of this paper highlighted that SO_2_ is an emerging signaling molecule in plants.

## Metabolism of SO_2_

### Anabolism of SO_2_

**Figure 1:**
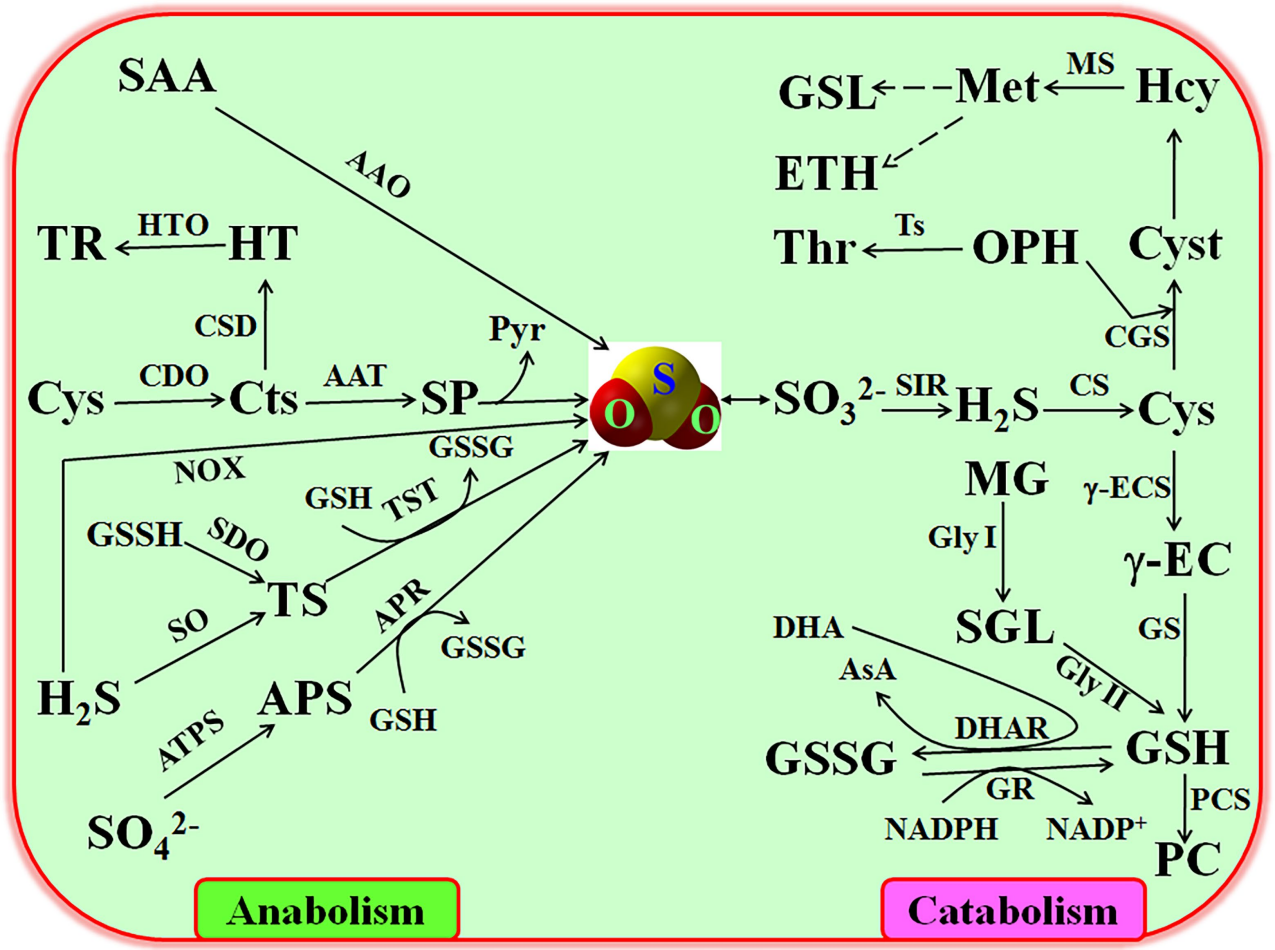
Anabolism and catabolism of sulfur dioxide (SO_2_) in plants. AAO, amino acid oxidase; APR, APS reductase; APS, adenosine 5′-phosphosulfate; ATPS, ATP sulfurylase; CDO, cysteine dioxygenase (CDO); CS, Cys synthetase; Cts, cysteine sulfinate; Cys, cysteine; Cyst, cystathionine; DHAR, dehydroascorbate reductase; γ-ECS, γ-glutamycysteine synthetase; Gly I, glyoxalase I; Gly II, glyoxalase II; GR, glutathione reductase; GS, GSH synthesis; GSH, glutathione; GSL, glucosinolate; GSSG, oxidized GSH; Hcy, homocystine; H_2_S, hydrogen sulfide; Met, methionine; MG, methylglyoxal; NOX, NADPH oxidase; OPH, O-phosphohomoserine; PC, phytochelatin; Pry, pyruvate; SAA, sulfur-containing amino acids; SGL, S-D-lactoylglutathione; SIR, sulfite reductase; SO, sulfide oxidase; SP, β-sulfinylpyruvate; Thr, threonine; Ts, threonine synthetase; TS, thiosulfate; TST, TS sulfurtransferase.

Similar to other signaling molecules, SO_2_ homeostasis in plants is maintained by its production (i.e., anabolism) and elimination (i.e., catabolism; Figure 1). In plants, the endogenous production of SO_2_ mainly roots in the reduction of ${\mathrm{SO}}_{4}^{2-}$ . ${\mathrm{SO}}_{4}^{2-}$ absorbed by roots is activated by ATP sulfurylase (ATPS), producing adenosine 5′-phosphosulfate (APS), which is transformed into ${\mathrm{SO}}_{3}^{2-}$ (main form of SO_2_ in solution) by APS reductase (APR) using glutathione (GSH) as reducing agent (Li et al., 2020). In addition, cysteine (Cys) can change into cysteine sulfinate (Cts) by the catalysis of cysteine dioxygenase (CDO), and then produce β-sulfinylpyruvate (SP), which automatically form SO_2_ without any enzyme catalysis and release pyruvate (Pyr; Yu et al., 2020; Huang et al., 2022). Also, SO_2_ can be released from hydrogen sulfide (H_2_S) and its derivant thiosulfate (TS) under the catalysis of NOX, sulfide oxidase (SO), and TS sulfurtransferase (TST), respectively (Li et al., 2009). Similarly, sulfur-containing amino acids (SAA) also can generate SO_2_ by the catalysis of amino acid oxidase (AAO; Rausch and Wachter, 2005). However, the detailed mechanisms of SO_2_-producing pathways in plant growth, development, and response to environmental stress need to be further dissected in the coming days.

### Catabolism of SO_2_

Analogue to its production, the scavenging of excessive SO_2_ is primarily achieved by the enzyme-catalysis pathways (Figure 1). Endogenous SO_2_ is easy to dissolve in water and produce ${\mathrm{SO}}_{3}^{2-}$ , which is in turn reduced into H_2_S by ${\mathrm{SO}}_{3}^{2-}$ reductase (SIR), followed by synthesizing Cys under the catalysis of Cys synthetase (CS; Hasanuzzaman et al., 2018; Li et al., 2020). Cys is a common precursor for biosynthesis of numerous biological molecules such as GSH, phytochelatin (PC), phytoalexins (PA), cystathionine (Cyst), homocystine (Hcy), methionine (Met), ethylene (ETH), and glucosinolate (GSL; Figure 1). GSH, as an important reducing agent in plant cells, which can be synthesized from Cys under the successive catalysis of γ-glutamycysteine synthetase (γ-ES) and GSH synthesis (GS; Yu et al., 2020; Huang et al., 2022). GSH and its oxidized form (GSSG) can mutually convert by dehydroascorbate reductase (DHAR) and glutathione reductase (GR) using dehydroascorbate and NADPH as electron acceptor and electron donor, respectively (Bartoli et al., 2017). Also, GSH can further synthesize PC to chelate heavy metal in plant cells, thus reducing the toxicity of heavy metal (Li et al., 2020). In addition to these, Cys can transform into other amino acids such as Cyst, Hcy, and Met (Li et al., 2009), further regulating the metabolism of amino acids in plants. These metabolic pathways are closely associated with the acquisition of stress tolerance in plants, also indicating the signaling role of SO_2_.

## Signaling Role of SO_2_

**Figure 2:**
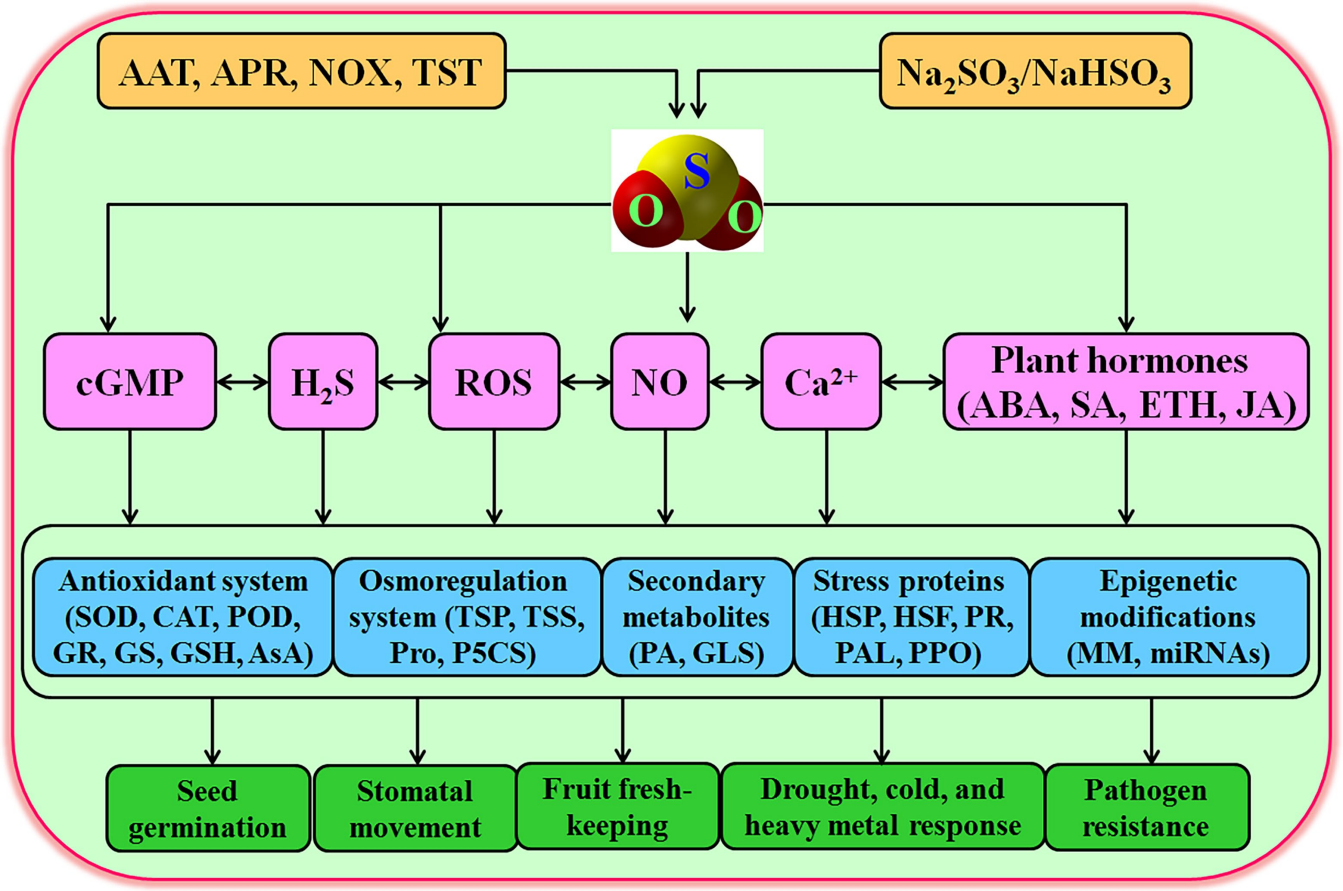
Signaling role of sulfur dioxide (SO_2_) in plants. AAT, aspartate aminotransferase; ABA, abscisic acid; APR, adenosine 5'-phosphosulfate reductase; AsA, ascorbic acid; CAT, catalase; cGMP, cyclic guanosine monophosphate; ETH, ethylene; GR, glutathione reductase; GS, glutathione synthetase; GSH, glutathione; GSL, glucosinolate; H_2_S, hydrogen sulfide; HSF, heat shock factor; HSP, heat shock proteins; JA, jasmonic acid; MM, methylation modification; NO, nitric oxide; NOX, NADPH oxidase; PA, phytoalexins; PAL, phenylalanine ammonia-lyase; P5CS, Δ^1^-pyrroline-5-carboxylate synthetase; POD, peroxidase; PPO, polyphenol oxidase; PR, pathogenesis-related proteins; Pro, proline; ROS, reactive oxygen species; SA, salicylic acid; SOD, superoxide dismutase; TSP, total soluble proteins; TSS, total soluble sugars; TST, thiosulfate sulfurtransferase.

Increasing evidences show that exogenous application of SO_2_ in a gas (mg m^−3^, fumigation) or solution (Na_2_SO_3_/NaHSO_3_, 3/1, irrigation) form could reprogramme the expression of thousands of genes. The physiological functions of these genes include antioxidant defense, osmotic adjustment, cell wall modification, and the synthesis of stress proteins (including heat shock proteins, HSP; and pathogen-related proteins, PR), secondary metabolites, and plant hormones (Li and Yi, 2012a,b, 2020; Zhao and Yi, 2014), which in turn modulated several physiological processes, such as seed germination, stomatal action, postharvest physiology, and plant response to environmental stresses, including drought, cold, heavy metal, and pathogen stresses (Table 1). In addition, the modulation of these physiological processes is involved in the interaction of SO_2_ and other signaling molecules, such as H_2_S, NO, ROS, cyclic guanosine monophosphate (cGMP), and plant hormones (Figure 2). In this section, the signaling role of SO_2_ in plants will be stated in detail.

**Table 1:** Examples of the role of SO_2_ signaling in plant growth and response to environmental stress.

**Species**	**Response**	**SO**_**2**_ **donor**	**Role**	**References**
Maize	Seed germination	0 ~ 5 mM Na_2_SO_3_/NaHSO_3_	Triggering NOX-dependent ROS signaling and elevating amylase activity	Guo et al., 2021
Wheat		0.1 mM Na_2_SO_3_/NaHSO_3_	Delaying programmed cell death (PCD) and triggering H_2_S/NO/ROS signaling	Sun et al., 2018
Barley		0.05 mM Na_2_SO_3_/NaHSO_3_	Delaying PCD and triggering H_2_S/NO/ROS signaling	Wang et al., 2017
Potato	Stomatal movement	0 ~ 5 mM Na_2_SO_3_/NaHSO_3_	Triggering H_2_S/NO signaling	Hu et al., 2014
*H. fulva*		0 ~ 5 mM Na_2_SO_3_/NaHSO_3_	Triggering PCD and NO/ROS/Ca^2+^ signaling	Wei et al., 2013
*Vicia faba*		0.25 ~ 6 mM Na_2_SO_3_/NaHSO_3_	Triggering NO/ROS/Ca^2+^ signaling	Yi et al., 2017
Longan	Fruit fresh-keeping	500 ~ 2,500 mg/L SO_2_	Triggering NOX-dependent ROS signaling	Joradol et al., 2019
Grape		200 μl/L	Driving AsA-GSH cycle and regulating sulfur metabolism	Zhang et al., 2022
Wheat	Drought	0 ~ 20 mg/m^3^ SO_2_	Triggering H_2_S signaling, synthesizing osmolyte, and activating antioxidant system	Li et al., 2021
Foxtail		30 mg/m^3^ SO_2_	Synthesizing osmolyte, triggering stomatal closure, and activating antioxidant system	Han et al., 2019
Arabidopsis	Cold	50 μM Na_2_SO_3_/NaHSO_3_ 30 mg/m^3^ SO_2_	Activating C-repeated binding factor (CBF) pathway and antioxidant system, synthesizing anthocyanin	Ma (2021)
Wheat	Cadmium	1 and 2 mM Na_2_SO_3_/NaHSO_3_	Triggering H_2_S signaling, synthesizing osmolyte, and activating antioxidant system	Hu et al., 2015
Foxtail		0.5 mM Na_2_SO_3_/NaHSO_3_	Triggering PCD, activating antioxidant system, and reducing metal transporters	Han et al., 2018
Wheat	Aluminium	1.2 mM Na_2_SO_3_/NaHSO_3_	Triggering H_2_S signaling, activating antioxidant system, and reducing aluminium accumulation	Zhu et al., 2015

### Seed Germination

Seed germination is the first and key stage of plant life cycle, which is sensitive to environmental stress, especially soil environment stress. Therefore, seed germination is the basis for crop production and vegetation recovery. In general, seed priming, especially chemical priming, can improve seed vigor, seed germination, and seedling viability, as well as the resistance of seedlings to adverse environments (Paul et al., 2022). In maize seeds, pretreatment with SO_2_ (1 mM) facilitated seed germination and increased seed vigor. In addition, the SO_2_-pretreated germinating seeds had higher NOX activity and ROS level, while NOX inhibitor, diphenyleneiodinium, inhibited ROS accumulation and germination and vigor of maize seeds pretreated with SO_2_ (Guo et al., 2021). Also, SO_2_ pretreatment up-regulated the expression and activity of α-amylase in germinating maize seeds (Guo et al., 2021). These data imply that SO_2_ might function as signaling molecule in facilitating the germination of maize seeds by mobilizing reserves *via* activating NOX-dependent ROS production.

In wheat seeds, pretreatment with 100 μM SO_2_ donor (NaHSO_3_/Na_2_SO_3_, 1/3 M/M) postponed PCD, restrained the coalescence of small protein storage vacuoles, and reduced the accumulation of ROS (e.g., hydrogen peroxide, H_2_O_2_; and superoxide anion, O_2_^–^) in aleurone cells pretreated with gibberellin (Sun et al., 2018). In addition, SO_2_-pretreated germinating seeds, compared to treatment with gibberelin alone, sustained higher activities of catalase (CAT), guaiacol peroxidase (POD), ascorbate peroxidase (APX), and superoxide dismutase (SOD), while had lower activities of lipoxygenase (LOX) and polyphenol oxidase (PPO; Sun et al., 2018). Also, SO_2_-pretreated aleurone layers induced the production of endogenous H_2_S and NO, whereas the supplement of cPTIO (NO scavenger) accelerated PCD in both SO_2_– and H_2_S-pretreated aleurone cells (Sun et al., 2018). These results indicate that SO_2_ can trigger H_2_S/NO signaling to delay PCD in wheat aleurone layers pretreated with gibberellin *via* activating antioxidant enzyme system.

Similarly, in barley (*Hordeum vulgare* L.) seeds, pretreatment with SO_2_ donor (NaHSO_3_/Na_2_SO_3_, 50 μM) alleviated PCD induced by gibberelin in aleurone layers in a concentration-dependent fashion (Wang et al., 2017). Additionally, SO_2_ pretreatment increased the activities of SOD, CAT, APX, POD, and glutathione reductase (GR), while weakened that of LOX, reduced the levels of H_2_O_2_, O_2_^–^, and malondialdehyde (MDA) in aleurone layers (Wang et al., 2017). Furthermore, SO_2_ pretreatment triggered the accumulation of endogenous H_2_S and NO in aleurone layers (Wang et al., 2017), similar to the results reported by Sun et al. (2018) in wheat. These data suggest that SO_2_ might regulate the germination of barley seeds by attenuating PCD *via* the interaction among H_2_S, NO, and ROS signaling.

### Stomatal Movement

Stomata is a major gate that plants exchange with carbon dioxide, oxygen, and water, its movement (i.e., opening and closure) influences photosynthesis, respiration, and transpiration, as well as plant resistance to environmental stress, especially drought stress (Jia and Zhang, 2008). Stomatal movement is strictly controlled by a signaling network composed of many second messengers, such as Ca^2+^, NO, ROS (mainly H_2_O_2_), H_2_S, and plant hormones (especially abscisic acid, ABA; ethylene, ETH; and cytokinin, CTK; Qi et al., 2018). Therefore, priming with signaling molecules can alter stomatal aperture and enhance the tolerance of plants to environmental stress. In sweet potato (*Ipomoea batatas*), treatment of epidermal strips with the different concentrations (0 ~ 5 mM) of Na_2_SO_3_/NaHSO_3_ solutions (SO_2_ donor) rapidly increased the levels of endogenous H_2_S and NO, and then induced stomatal closure in a dose-dependent manner (Hu et al., 2014). In addition, the stomatal closure induced by SO_2_ was reversed by hypotaurine (H_2_S scavenger) and cPTIO (NO scavenger; Hu et al., 2014), indicating that the SO_2_-triggered stomatal closure might be mediated by the H_2_S and NO signaling pathways. Also, in broad bean (*Vicia faba* L.), low concentrations (0.0001 ~ 0.1 μM) of sulfurous acid (H_2_SO_3_, as SO_2_ donor) facilitated stomatal opening by reducing the level of endogenous ABA to antagonize its action (Taylor et al., 1981). Adversely, a high concentration (10 μM) of H_2_SO_3_ inhibited stomatal opening by increasing the level of endogenous ABA (Taylor et al., 1981).

In *Hemerocallis fulva*, the epidermal strips treated with the different concentrations (1 ~ 5 mM) of SO_2_ donor (Na_2_SO_3_/NaHSO_3_) were found to reduce the guard cells vigor and induce cell death in a concentration-dependent manner (Wei et al., 2013). The SO_2_-induced death cells exhibited the features of apoptosis (i.e., nuclear condensation, nuclear elongation, and DNA fragmentation), indicating SO_2_ could trigger PCD in guard cells. Additionally, the levels of endogenous NO, ROS, and Ca^2+^ were increased by SO_2_, while reduced by NO scavenger (cPTIO), nitrate reductase (NO-producing enzyme) inhibitor (NaN_3_), ROS scavengers (ascorbic acid, AsA; and CAT), Ca^2+^ chelating agent (EGTA), and plasma membrane Ca^2+^ channel blocker (LaCl_3_), thus decreasing cell death (Wei et al., 2013). Also, AsA treatment decreased the levels of NO and Ca^2+^ compared with the SO_2_ treatment alone, whereas NaN_3_ treatment decreased ROS and Ca^2+^ levels, but LaCl_3_ treatment had no significant effect on NO and ROS levels (Wei et al., 2013). These data imply that SO_2_ could induce PCD in guard cells by intertwining NO, ROS, and Ca^2+^ signaling pathways in *H. fulva*, which might be a major cause for SO_2_-induced stomatal closure.

Similarly, in *Vicia faba*, SO_2_ hydrates (Na_2_SO_3_/NaHSO_3_) induced guard cell death in a dose-dependent fashion (ranging from 0.25 to 6 mM; Yi et al., 2017). Meanwhile, SO_2_ induced an increase in the level of endogenous NO, H_2_O_2_, and Ca^2+^ in guard cells of *Vicia faba*, similar to the data reported by Wei et al. (2013) in *H. fulva*. In addition, treatment with exogenous NO donor enhanced the toxicity of SO_2_, whereas NO scavenger (cPTIO) and synthesis inhibitors (L-NAME and tungstate) weakened SO_2_ toxicity (Yi et al., 2017). Likewise, the toxicity of SO_2_ was also blocked by ROS scavenger (AsA and CAT), Ca^2+^ chelating agent (EGTA), and Ca^2+^ channel inhibitor (LaCl_3_; Yi et al., 2017). Also, treatment with both cPTIO and AsA reversed SO_2_-induced increase in Ca^2+^ level in guard cells, while cPTIO and AsA treatment alone blocked SO_2_-induced H_2_O_2_ and NO accumulation (Yi et al., 2017). These data suggest that SO_2_ toxicity might be achieved by joint action of NO, ROS, and Ca^2+^ signaling in guard cells of *Vicia faba*, further supporting the hypothesis proposed by Wei et al. (2013). In addition to stomatal movement, Haworth et al. (2012) reported that SO_2_ could change stomatal density (SD), stomatal index (SI), and SD/SI ratio in *Lepidozamia hopei*, *Lepidozamia peroffskyana*, *Ginkgo biloba*, *Nageia nagi*, *Podocarpus macrophyllus*, *Araucaria bidwillii*, and *Aagathis australis*.

### Fruit Fresh-Keeping

Shelf-life of the fruits and vegetables determines their quality and economic value. To lengthen the shelf-life, numerous physical and chemical methods are used in the process of fruits and vegetables storage (Joradol et al., 2019). The physical methods are involved in water, fertilizer, gas (e.g., oxygen and carbon dioxide), heat (temperature), and light; while chemical methods are mainly fresh-keeping agent treatments (Zhang et al., 2022). In Longan (*Dimocarpus longan* Lour. cv. Daw) fruits, SO_2_ fumigation (500 ~ 2,500 mg L^−1^) reduced pericarp browning, lengthened shelf-life, and improved the quality of fruits by enhancing antioxidant capacity compared with the control without SO_2_ fumigation (Joradol et al., 2019). In addition, the fruits fumigated with SO_2_ had a higher content of endogenous H_2_O_2_, which reached a maximum within 6 ~ 12 h. Also, SO_2_ treatment up-regulated the gene expression of NOX and SOD (Joradol et al., 2019). These data indicate that SO_2_ fumigation can induce the NOX-dependent H_2_O_2_ signaling, which in turn enhance the antioxidant capacity, thereby lengthening the shelf-life of fruits.

Similarly, in table grapes, SO_2_ treatment (200 μl L^−1^) inhibited fruit decay and reduced the levels of H_2_O_2_ and MDA compared to the control without SO_2_ treatment (Zhang et al., 2022). Additionally, SO_2_ treatment up-regulated the expression of *VvSiR, VvSAT1*, *VvSAT2*, and *VvOASTL*, which in turn increased the activities of sulfite reductase, serine acetyltransferase, and *O*-acetylserine (thiol)-lyase, as well as the content of Cys (Zhang et al., 2022). Likewise, the gene expression level, enzyme activity, and antioxidant content related to AsA-GSH cycle was enhanced by SO_2_ treatment in grapes. Also, the expression of *VvGS* (GSH synthetase) was up-regulated by SO_2_ treatment in table grapes, while the transcription level of *VvHPCA1-4* and *VvHPCA3* (evaluating the degree of oxidative damage) was down-regulated (Zhang et al., 2022). These data indicate that SO_2_ can lengthen the shelf-life of table grapes by maintaining H_2_O_2_ homeostasis to reduce postharvest oxidative damage *via* driving the AsA-GSH cycle.

### Drought Response

Drought stress commonly leads to osmotic and oxidative stress due to the shortage of water and the overaccumulation of ROS in plant cells. The approaches alleviating osmotic and oxidative stresses can boost the resistance of plants to drought stress (Salehi-Lisar and Bakhshayeshan-Agdam, 2016). In wheat seedlings, under drought stress, pretreatment with SO_2_ (0 ~ 20 mg m^−3^) improved the survival percentage and relative water content (Li et al., 2021), indicating that SO_2_ could increase drought tolerance. The further experiments showed that SO_2_ pretreatment improved the content of proline (Pro) and activities of SOD and POD, which in turn reduced the accumulation of H_2_O_2_ and MDA in drought-treated wheat seedlings. In addition, SO_2_ pretreatment down-regulated the expression of *TaNAC69* (transcription factor gene), while the expression of other transcription factor genes (*TaERF1* and *TaMYB30*) insignificantly changed but maintained a higher levels in wheat seedlings under drought stress conditions (Li et al., 2021). Also, SO_2_ pretreatment induced an increase in H_2_S in wheat seedlings under drought stress, while H_2_S scavenger hypotaurine decreased the activities of SOD and POD, as well as the expression of transcription factor genes, followed by increasing the accumulation of H_2_O_2_ and MDA, returning to the level of drought treatment alone (Li et al., 2021). These data suggest that SO_2_ can increase the drought tolerance of wheat seedlings by accumulating osmolytes and activating antioxidant enzmyes *via* H_2_S signaling pathway.

In like manner, in foxtail millet seedlings, SO_2_ fumigation (30 mg m^−3^) decreased stomatal apertures and leaf transpiration rate, which in turn improved the relative water content in the leaves of drought-stressed seedlings (Han et al., 2019), thus improving the drought tolerance of seedlings. Additionally, SO_2_ pretreatment increased the activity of Δ^1^-pyrroline-5-carboxylate synthetase (P5CS), reduced that of Pro dehydrogenase (ProDH), and corresponding gene expression, followed by accumulating Pro in the leaves of drought-stressed seedlings (Han et al., 2019). Moreover, application of SO_2_ up-regulated the gene expression of CAT and POD and increased the activities of corresponding enzymes in the leaves of drought-stressed plants, which in turn alleviated drought-induced oxidative damage (i.e., decreasing MDA content) by scavenging H_2_O_2_ (Han et al., 2019). These results imply that SO_2_ fumigation can increase drought tolerance in foxtail millet seedlings by combined effect of stomatal closure, Pro accumulation, and antioxidant defense. Similarly, under drought stress, SO_2_ pre-exposure (30 mg m^−3^) increased SOD, POD, and O-acetylserine(thio)lyase (OASTL) activities and GSH, Cys, and nonprotein thiol (NPT) contents in Arabidopsis plants, which in turn alleviated oxidative stress (i.e., reducing H_2_O_2_ and MDA accumulation; Li and Yi, 2022). Meanwhile, SO_2_ increased Pro level by up-regulating gene expression and activity of P5CS and down-regulating that of ProDH, followed by reducing water loss, stomatal conductance, and transpiration rate, thus increasing net photosynthetic rate, water use efficiency, and photosynthetic pigment contents (Li and Yi, 2022), indicating that SO_2_ can improve the tolerance of Arabidopsis plants to drought stress.

### Cold Response

Cold stress includes chilling (above freezing point, resulting in chilling injury) and freezing stress (below freezing point, leading to freezing injury). Both chilling and freezing stress can trigger osmotic and oxidative stresses (Ritonga and Chen, 2020). Therefore, the enhanced osmoregulation and antioxidant capacity by chemical priming are closely related with low temperature stress tolerance in plants. Presoaking of seeds with the different concentrations of (0, 10, 25, 50, and 100 μM) SO_2_ alleviated the cold-induced growth inhibition. Among concentrations, 50 μM SO_2_ presoaking was the most efficient (Ma, 2021). Similarly, pretreatment of *Arabidopsis thaliana* seeds with 50 μM SO_2_ boosted the cold resistance of *A. thaliana* seedlings. Otherwise, SO_2_ presoaking up-regulated the expression of *AtCAT3* and *AtPOD*, and increased the activities of corresponding CAT and POD, which in turn reduced the level of endogenous H_2_O_2_ (Ma, 2021). Meanwhile, 50 μM SO2 pretreatment up-regulated the expression of genes related to anthocyanin synthesis (i.e., *AtPAL2, AtCHS, AtCHI, and AtF3H*), followed by promoting the synthesis of anthocyanin. Interestingly, SO_2_ pretreatment also activated the expression of genes (i.e., *AtICE1, AtICE2, AtCBF1, AtCBF2, AtCBF3, AtCOR15a, and AtCOR15b*) involved in C-repeated binding factor (CBF) signaling pathways, mainly cold-response signaling pathways (Ma, 2021).

Parallelly, SO_2_ (30 mg m^−3^) exposure reduced stomatal aperture and ROS accumulation by enhancing POD activity under cold stress (Ma, 2021). Also, SO_2_ exposure promoted Pro accumulation by increasing P5CS activity and lowering PDH activity as well as activated the gene expression of CBF signaling pathways (Ma, 2021), thus improving the cold adaptability of *Arabidopsis thaliana*. Similarly, exposure of *Arabidopsis thaliana* (Col-0) to the different concentrations of SO_2_ increased O_2_^–^ generation rate and H_2_O_2_ content in Arabidopsis shoots, and up-regulated the gene expression of POD, SOD, and glutathione peroxidase (GPX), as well as increased the activity of corresponding enzymes and the content of GSH. Additionally, SO_2_ exposure increased isoenzymatic isoforms of SOD (FeSOD and Cu/ZnSOD) and POD, while decreased CAT isoforms (CAT2 and CAT3; Li and Yi, 2012b). Similarly, in Arabidopsis, SO_2_ exposure (30 mg m^−3^) down-regulated the expression of miR398, which in turn increased the transcript level of its target genes Cu/Zn-SOD (*CSD1* and *CSD2*) and increased the activity of SOD (Li et al., 2017). Similarly, SO_2_ up-regulated the expression of miR395 expression, followed by decline in the transcript level of its target genes, ATP sulfurylases (*APS3* and *APS4*) and sulfate transporter (*SULTR2;1*), implying miR398 and miR395 participate in the resistance of Arabidopsis plants to oxidative stress induced by SO_2_ exposure. These data suggest that SO_2_-induced ROS act as a signal to trigger plant defense response.

### Heavy Metal Response

Heavy metals, such as cadmium (Cd), mercury, arsenic, and aluminum, have become the major pollutants with the development of modern agriculture and industry. Heavy metals can disturb cellular metabolism, inhibit seed germination and plant growth, and even influence on human health by entering into food chain (Ghori et al., 2019). Therefore, heavy metal pollutions have become a huge challenge for crop production, food safety, and human health. How to improve the resistance of crop plants to heavy metal stress and reduce its endogenous accumulation is an urgent issue. In wheat seeds, Cd-inhibited seed germination, while the inhibiting effects were alleviated by exogenous application of SO_2_ (Na_2_SO_3_/NaHSO_3_ (3/1) solution as SO_2_ donor) in a concentration-dependent manner and the optimal concentration was between 1 and 2 mM (Hu et al., 2015). Additionally, SO_2_ donor pretreatment enhanced the activities of amylase and esterase, which in turn resulted in the accumulation of total soluble sugars (TSS) and total soluble protein (TSP) in germinating seeds under Cd stress (Hu et al., 2015). Also, SO_2_ pretreatment reduced the overproduction of MDA, H_2_O_2_, and O_2_^–^, as well as the loss of plasma membrane integrity of the radicle tips of seedlings under Cd stress (Hu et al., 2015). Further experiment data showed that SO_2_ increased the activities of POD, APX, SOD, and CAT, lowered level of LOX in germinating wheat seeds (Hu et al., 2015). Interestingly, compared with the control, SO_2_ pretreatment increased the level of endogenous H_2_S in germinating wheat seeds (Hu et al., 2015). These data suggest that SO_2_ can promote the germination of wheat seeds under Cd stress by mobilizing reserves and activating antioxidant system *via* H_2_S signaling pathway.

In foxtail millet seedlings, application of SO_2_ derivatives (0.5 mM) reduced the Cd-inhibited seedling growth and Cd-induced oxidative damage (i.e., reducing MDA accumulation) in the leaves of seedlings (Han et al., 2018). Additionally, SO_2_ treatment enhanced the activities of CAT and SOD and drove AsA-GSH cycle, which in turn reduced the accumulation of Cd-elicited O_2_^–^ and H_2_O_2_ in the leaves of seedlings (Han et al., 2018). Furthermore, SO_2_ application increased the contents of GSH and phytochelatin (PC) by promoting sulfur assimilation, followed by enhancing Cd-detoxification capacity in seedlings. Further experiments showed that SO_2_ derivatives down-regulated the expression of genes related to Cd uptake and translocation (i.e., *NRAMP1*, *NRAMP6*, *IRT1*, *IRT2*, *HMA2*, and *HMA4*; Han et al., 2018), thus reducing Cd accumulation in the shoots and roots of Cd-stressed seedlings.

Similarly, in wheat seeds, pretreatment with SO_2_ donor (NaHSO_3_/Na_2_SO_3_, 1/3) at 1.2 mM increased the activities of POD, CAT, and APX, and lowered that of LOX, and reduced the accumulation of O_2_^–^, H_2_O_2_, and MDA in germinating seeds under aluminum stress (Zhu et al., 2015). As expected, SO_2_ pretreatment increased the level of endogenous H_2_S in wheat seeds, while reduced the content of ROS, which in turn maintained the integrity of biomembrane and reduced aluminum accumulation in wheat seedling radicles (Zhu et al., 2015). Also, SO_2_ pretreatment down-regulated the expression of alumium-responsive genes (i.e., *TaWali1*, *TaWali2*, *TaWali3*, *TaWali5*, *TaWali6*, and *TaALMT1*) in seedling radicles under aluminum stress (Zhu et al., 2015). These data indicate that SO_2_ can improve seed germination of wheat under aluminum stress by enhancing antioxidant capacity and reducing the accumulation of aluminum *via* H_2_S signaling pathway.

### Pathogen Response

Besides enhancing abiotic stress tolerance, exogenously applied SO_2_ also can improve the resistance of plants to biotic stress (Zhao and Yi, 2014). In *Arabidopsis* plants, SO_2_ pre-exposure (30 mg m^−3^) increased the resistance to *Botrytis cinerea* (Xue and Yi, 2018). Further experiments showed that SO_2_ pretreatment up-regulated the expression levels of the defense-related genes (*PAL*, *PPO*, *PR2*, and *PR3*), which encode phenylalanine ammonia-lyase (PAL), polyphenol oxidase (PPO), β-1,3-glucanase (BGL), and chitinase (CHI), and increased the activities of PAL, PPO, BGL, and CHI (Xue and Yi, 2018). Also, SO_2_ pre-exposure increased the transcript levels of microRNAs (MIR393, MIR160, and MIR167), correspondingly decreased the gene expression of their targets involved in the auxin signaling pathway. Adversely, the expression levels of the primary auxin-response genes (*GH3-like*, *BDL/IAA12*, and *AXR3/IAA17*) were down-regulated by SO_2_ pre-exposure in *Arabidopsis* plants (Xue and Yi, 2018). These data imply that SO_2_ increases the disease resistance of Arabidopsis plants to *Botrytis cinerea* by enhancing the defense-related gene expression and enzyme activity as well as suppressing the auxin signaling pathway mediated by miRNA.

In addition, Arabidopsis plants, the transcriptome analysis identified that SO_2_ fumigation (30 mg m^−3^) led to the change in the expression of 2,780 genes, the genes involved in biotic stress resistance, ROS production and scavenging, and sulfur assimilation were up-regulated (Zhao and Yi, 2014). Likewise, Li and Yi (2012b) reported that SO_2_ (30 mg m^−3^) treatment triggered the different expression of 2,780 genes, the functions of which were mainly involved in signal transduction, transcription regulation, molecular structure, transport, binding, and metabolism. Many different expression genes encoding antioxidant enzymes (POD, GPX, and SOD), heat shock proteins (HSP), pathogenesis-related (PR) proteins, and cytochrome P450 were up-regulated by SO_2_ in Arabidopsis shoots (Li and Yi, 2012b). In Arabidopsis, Li and Yi (2012a), using Affymetrix GeneChip technology, found that the expression of 494 genes was significantly changed by SO_2_ exposure (30 mg m^−3^), they encoded antioxidant enzymes (e.g., GST, POD, and thioredoxin), HSP, and PR, as well as were involved in the ETH signaling pathway, phenylpropanoid pathway, and cell wall modification. Similarly, SO_2_ exposure enhanced the production of ROS, as signaling molecule, increased the activities of SOD, POD, glutathione peroxidase (GPX), and GST in Arabidopsis plants (Li and Yi, 2012a). Also, in fresh table grapes (*V. vinifera* L. “Crimson Seedless”), transcriptomics approaches also found that SO_2_ treatment (140 μl L^−1^) up-regulated the expression of enzymes genes related to sulfur-metabolizing enzymes (especially directing towards chelation and conjugation), redox homeostasis, and plant hormones (e.g., auxin, AUX; ethylene, ETH; and jasmonic acid, JA) signaling pathways (Giraud et al., 2012). Generally, salicylic acid (SA) level is closely related to plant disease resistance. In Arabidopsis thaliana plants, Hao et al. (2011) found that the *snc1* mutants (with high SA content) had higher tolerance to SO_2_ than *nahG* plants (with low SA content), implying that endogenous SA and signaling might play an essential role in plant responses to SO_2_ stress. These studies further support the fact that SO_2_ increases disease resistance in Arabidopsis plants.

## Conclusion and Perspectives

Mounting evidences show that SO_2_ can not only regulate seed germination, stomatal movement, and postharvest physiology, but also plants respond to environmental stresses, such as drought, cold, and heavy metal, and pathogen stresses. Numerous studies identified that SO_2_ meets the requirements of signaling molecules in plants, emerging as a novel signaling role in many plant physiological processes. SO_2_ as a novel signaling molecule can exert its physiological functions either alone or interaction with other signaling molecules, such as Ca^2+^, H_2_O_2_, NO, H_2_S, cGMP, and plant hormones (e.g., ABA, SA, ETH, and JA; Figure 2). SO_2_ can enhance antioxidant system (e.g., SOD, CAT, POD, APX, GR, GSH, and AsA) and osmoregulation system (e.g., Pro, TTP, TSS, and P5CS), drive secondary metabolism (e.g., GLS and PA accumulation), synthesize stress proteins (e.g., HSP, HSF, PR, PAL, and PPO), and modulate epigenetic modifications (e.g., DNA methylation modification (MM) and miRNAs), thus regulating several plant physiological functions (Figure 2). Though, the signaling role of SO_2_ in plants has been verified in a large amount of physiological processes, numerous open questions still need to be further answered in more detail in the coming days. In mammalian cells, the AAT/SO_2_ pathway is contributed to SO_2_ signaling; however, in plants, besides the APR/ ${\mathrm{SO}}_{3}^{2-}$ pathway (Figure 1), the detailed metabolic pathways of SO_2_ are waiting for being further expounded. Correspondingly, the knowledge on the exact concentrations of SO_2_ in plant cells and subcellular structures as well as its receptors/sensors remains to be found. Though SO_2_ could improve multiple stress tolerance, whether SO_2_ can induce the tolerance of plants to heat, salt, and flooding stresses and the underlying mechanisms requires to be further explored. In addition, the signaling interaction of SO_2_ with Ca^2+^, ROS, NO, H_2_S, methylglyoxal, and cyclic nucleotide in plants under both physiological and stress conditions is necessary to be uncovered. With the development of omics, the effects of SO_2_ on transcriptome, metabolome, proteome, and phenome in plants have to be settled urgently.

## Author Contributions

Z-GL conceived, designed, and wrote the manuscript, while X-EL and H-YC wrote the anabolism and catabolism of SO_2_, respectively. All authors contributed to the article and approved the submitted version.

## Funding

This research was funded by the National Natural Science Foundation of China (32160065 and 31760069).

## Conflict of Interest

The authors declare that the research was conducted in the absence of any commercial or financial relationships that could be construed as a potential conflict of interest.

## Publisher’s Note

All claims expressed in this article are solely those of the authors and do not necessarily represent those of their affiliated organizations, or those of the publisher, the editors and the reviewers. Any product that may be evaluated in this article, or claim that may be made by its manufacturer, is not guaranteed or endorsed by the publisher.
